# The Editorial in July Number

**Published:** 1893-10

**Authors:** Edward Payson Small

**Affiliations:** Philadelphia


### THE EDITORIAL IN THE JULY NUMBER.

EDITOR OF THE HOMŒOPATHIC PHYSICIAN:—Thank you for the editorial in the July number. It seems to me an excellent presentation of the exact truth. What an admirable tract it would make.

All do not possess equal powers of expression, but we may all follow and work in the path opened providentially by Hahnemann for the good of humanity. There is "a good time coming" for Homœopathy, for now is but the dawn of the new age. With "Sojourner Truth" we ask is "God dead"?

Fraternally,
EDWARD PAYSON SMALL, M. D.

PHILADELPHIA, August 6th, 1893.

